# *Pseudomonas aeruginosa* Airway Infection Recruits and Modulates Neutrophilic Myeloid-Derived Suppressor Cells

**DOI:** 10.3389/fcimb.2016.00167

**Published:** 2016-11-29

**Authors:** Hasan H. Öz, Benyuan Zhou, Pina Voss, Melanie Carevic, Carolin Schroth, Nina Frey, Nikolaus Rieber, Andreas Hector, Dominik Hartl

**Affiliations:** ^1^Children's Hospital and Interdisciplinary Center for Infectious Diseases, University of TübingenTübingen, Germany; ^2^Department of Pediatrics, Kinderklinik München Schwabing, Klinikum Schwabing, StKM GmbH und Klinikum rechts der Isar, Technische Universität MünchenMunich, Germany; ^3^Roche Pharma Research and Early Development, Immunology, Inflammation and Infectious Diseases (I3) Discovery and Translational Area, Roche Innovation Center BaselBasel, Switzerland

**Keywords:** MDSCs, bacteria, *Pseudomonas*, cystic fibrosis, CFTR, lung, T-cells

## Abstract

*Pseudomonas aeruginosa* is an opportunistic pathogen that causes infections mainly in patients with cystic fibrosis (CF) lung disease. Despite innate and adaptive immune responses upon infection, *P. aeruginosa* is capable of efficiently escaping host defenses, but the underlying immune mechanisms remain poorly understood. Myeloid-derived suppressor cells (MDSCs) are innate immune cells that are functionally characterized by their potential to suppress T- and natural killer (NK)-cell responses. Here we demonstrate, using an airway *in vivo* infection model, that *P. aeruginosa* recruits and activates neutrophilic MDSCs, which functionally suppress T-cell responses. We further show that the CF gene defect (CF transmembrane conductance regulator, CFTR) modulates the functionality, but not the recruitment or generation of neutrophilic MDSCs. Collectively, we define a mechanism by which *P. aeruginosa* airway infection undermines host immunity by modulating neutrophilic MDSCs *in vivo*.

## Introduction

Infections with *Pseudomonas aeruginosa*, an opportunistic gram-negative bacterium, represent a major cause of morbidity and mortality in patients with cystic fibrosis (CF) lung disease, chronic obstructive pulmonary disease (COPD), ventilated patients, and patients undergoing immunosuppression (Williams et al., 2010; Hartl et al., 2012). Upon infection, *P. aeruginosa* activates the innate immune system and induces a rapid recruitment of neutrophilic cells to the site of inflammation, followed by activation of the adaptive response characterized by T-cell infiltration (Hartl et al., 2012). Despite a substantial and sustained presence of phagocytic and lymphocytic immune cells at the infected compartment, the host is not able to efficiently eliminate *P. aeruginosa*, particularly in pulmonary disease conditions, such as CF (caused by mutations in the CF transmembrane conductance regulator, CFTR, gene) or chronic obstructive pulmonary disease (COPD, caused by cigarette smoke; Hartl et al., 2012; Mall and Hartl, 2014; Yonker et al., 2015). The underlying immune mechanisms remained poorly understood.

Myeloid-derived suppressor cells (MDSCs) are innate immune cells that are functionally characterized by their potential to suppress T- and natural killer (NK)-cell responses (Gabrilovich and Nagaraj, 2009; Gantt et al., 2014). MDSCs can be sub-divided into neutrophilic and monocytic MDSCs as defined by surface marker profiles. Rieber et al. demonstrated in a previous study that neutrophilic MDSCs accumulate in patients with CF infected with *P. aeruginosa* (Rieber et al., 2013). We further showed that neutrophilic MDSCs are clinically relevant in *P. aeruginosa* infected CF patients, because percentages of neutrophilic MDSCs correlated with lung function in those patients. However, the mechanisms by which *P. aeruginosa* airway infection regulates neutrophilic MDSCs *in vivo* remained elusive.

Here, we used a well-established *P. aeruginosa* airway infection model (Munder and Tummler, 2014; Hector et al., 2015) to investigate the mechanisms by which *P. aeruginosa* skews host immunity *in vivo*. Our studies demonstrate that (i) *P. aeruginosa* airway infection triggers the recruitment of neutrophilic, but not monocytic MDSCs, (ii) *P. aeruginosa* infection enhances the suppressive capacity of neutrophilic MDSCs, and (iii) CFTR partially overlaps with *P. aeruginosa* in modulating neutrophilic MDSCs.

## Materials and methods

### Ethics statement

All animal studies were reviewed and approved by the Regierungspräsidium Tübingen, Tübingen, Germany (approval ID: K4/12), and were carried out according to the guidelines of the German law of protection of animal life.

### Bacteria

*P. aeruginosa* wild type strains (PAO1) were used as published previously by our group (Hector et al., 2015). Strains of the culture collection were streaked on agar plates and incubated at 37°C overnight. Colonies were then inoculated into lysogeny broth overnight. The next day, a 1:10 dilution in lysogeny broth was performed and bacteria were cultured at 37°C for 4 h. The optical density was measured at 600 nm.

### Mouse models

Mice were bred at the animal facility of the Institute of Pharmacology (Tübingen). C57Bl/6J or *Cftr*^−/−^ [*Cftr*^*tm*1*Unc*^–*Tg(FABPCFTR)1Jaw/J*] mice were used. *Cftr*^−/−^ mice were compared with age- and background strain-matched *Cftr*^+/+^ littermates. The mouse model of acute pulmonary *P. aeruginosa* infection was performed as published by our group (Hector et al., 2015). Mice were infected with 2 × 10^6^ or 4 × 10^6^ CFU of *P. aeruginosa* (PAO1) utilizing previously established procedures (Hector et al., 2015). Intranasal applications were carried out under antagonizable anesthesia. Briefly, an inoculum of 2 × 10^6^ and 4 × 10^6^ CFU were administered intranasally (50 μl/nostril). After infection, body weight was monitored once a day over 1 week. For FACS analysis mice were sacrificed 16 h after infection.

### MDSC *in vitro* generation

Bone marrow cells and splenocytes were isolated from *Cftr*^+/+^ and *Cftr*^−/−^ mice and cultured in RPMI1640 (Merck Millipore) with supplements, in detail 10% fetal bovine serum (Sigma), 100 U/ml Penicillin-Streptomycin (Merck Millipore) each, 2 mM L-Glutamine (Merck Millipore), 10 mM HEPES (Merck Millipore). MDSC generation was induced by addition of 40 ng/ml recombinant mouse GM-CSF (Biolegend) and 40 ng/ml recombinant mouse IL-6 (Biolegend) as published by Marigo et al. (2010). Cells were either fed with fresh media and cytokines on d3 and d6 or collected and analyzed by flow cytometry at d3, d6, and d10. For suppression assays cells were collected on d6.

### MDSC characterization, isolation, and adoptive transfer

Murine MDSCs were phenotypically characterized as described previously (Rieber et al., 2015) by using CD11b, Ly6G (neutrophilic MDSCs), and Ly6C (monocytic MDSCs) markers followed by T-cell suppression assays to distinguish them from non-suppressive neutrophilic or monocytic effector cells, respectively. Flow cytometry was performed on a FACS Calibur (BD). Murine MDSCs were isolated from different organs/tissues/fluids as described previously (Rieber et al., 2015) using MACS (MDSC isolation kit, Miltenyi). For adoptive transfer experiments, CD11b^+^Ly6G^+^ neutrophilic MDSCs were isolated from the bone marrow of healthy female C57Bl6/J wildtype mice by MACS (MDSC isolation kit, Miltenyi Biotec, Germany). Transfer was performed by transferring 8–10 × 10^6^ neutrophilic MDSCs per animal into 8–12 weeks old female C57Bl6/J wildtype mice via lateral tail vein injection. 24 h after the neutrophilic MDSC transfer, mice were infected with *P. aeruginosa* (PAO1) as described above.

### T-cell suppression assays

T-cell suppression assays were performed as described previously (Rieber et al., 2015) using the CFSE method according to the manufacturer's protocol (Invitrogen). In brief, CD11b^+^Ly6G^+^ neutrophilic MDSCs were isolated from different organs/tissues/fluids by using MACS (MDSC isolation kit, Miltenyi Biotec, Germany) and were co-cultured for 3 days (37°C, 5% CO_2_) with MACS sorted CFSE stained CD4^+^ T-cells from splenocytes at MDSC: T-cell ratios 1:1, 1:2, 1:4, and 1:8 in RPMI1640 with supplements as mentioned before. The number of T-cells per well was kept at 10^5^ and MDSCs were added accordingly. T-cell proliferation was stimulated with CD3/CD28-beads (mouse T-cell activation kit, Miltenyi Biotec, Germany) and recombinant mouse IL-2 (50 U/ml, Biolegend). CFSE-fluorescence intensity was analyzed by flow cytometry to determine the percentage of polyclonally proliferated T-cells. For the graphs the data was normalized to the proliferation of the stimulated control T-cell proliferation (without addition of MDSCs).

### BALF

Bronchoalveolar lavage was extracted through the trachea with 2 ml PBS. Living BAL cells were counted using trypan blue dye exclusion. After erythrocyte lysis with ACK lysis solution, cells were incubated with an Fc receptor block (1 μg/1 × 10^6^ cells; BD Bioscience) to reduce nonspecific antibody binding.

### Flow cytometry

The panel of antibodies used to stain BALF, lung, spleen, or bone marrow cells included CD11b (clone M1/70), Ly6G (clone 1A8), and Ly6C (clone AL-21; all from BD Biosciences). Flow cytometry was performed using BD FACS Calibur (BD Bioscience), and data were analyzed with FlowJo software.

### Statistics

Statistical analysis was done using GraphPad Prism 6.0 (Graph Pad Software, La Jolla, CA, USA). Differences between the groups were determined by Students' *t*-test. A *p* < 0.05 was considered to be significant.

## Results

### Pseudomonas infection triggers neutrophilic MDSC recruitment

**Figure 1:**
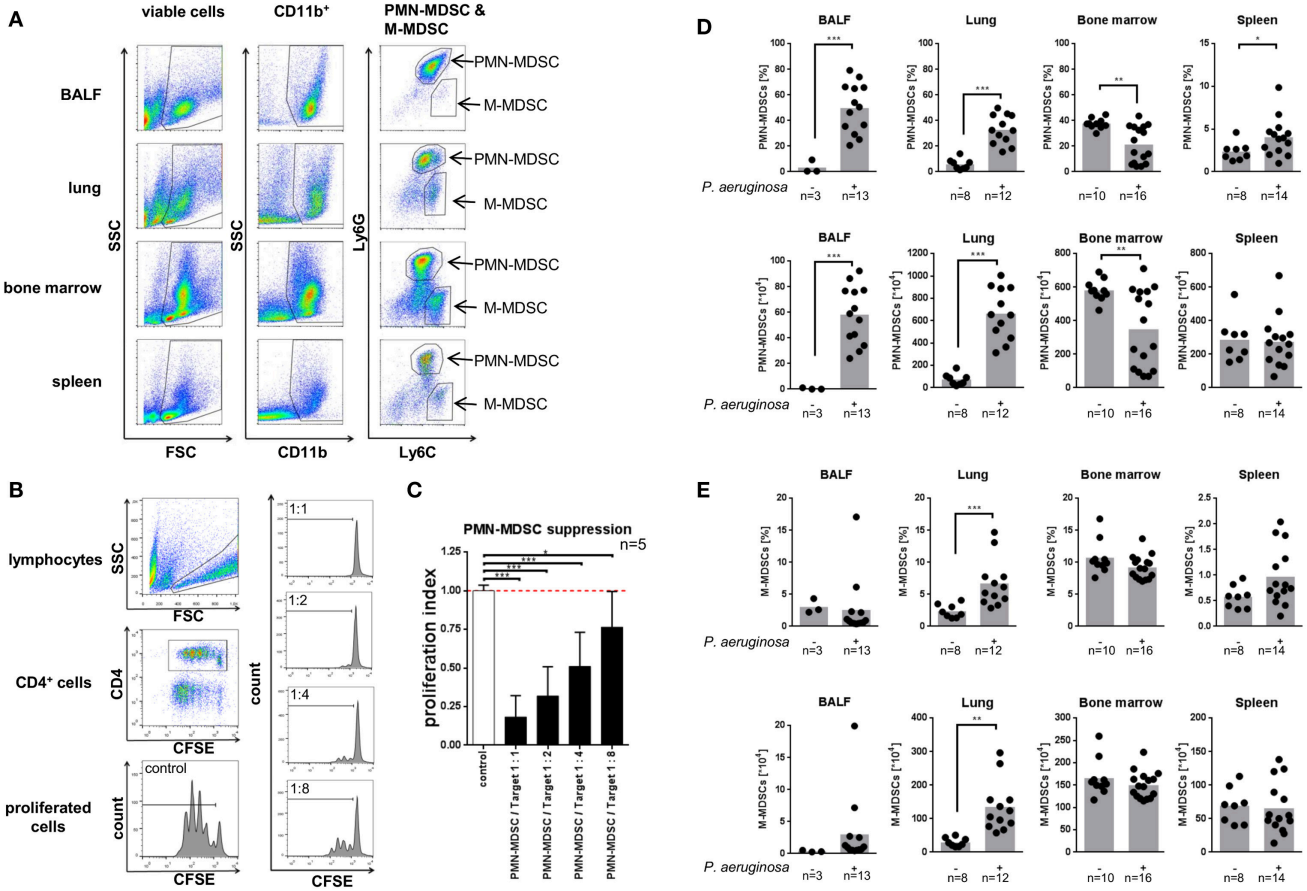
*****Pseudomonas aeruginosa*** airway infection recruits MDSCs. (A)** Neutrophilic (PMN–) MDSCs and monocytic (M–) MDSCs in BALF, lung, bone marrow, and spleen were sequentially gated based on FSC/SSC, CD11b, Ly6G, and Ly6C. PMN-MDSCs were assessed as CD11b^+^Ly6G^+^Ly6C^intermediate^, M-MDSCs as CD11b^+^Ly6C^+^Ly6G^low^. **(B)** Left panel: Gating strategy to assess CD4^+^ T-cell proliferation using CFSE staining and flow cytometry. CD4^+^ cells were gated from the lymphocyte population in FSC/SSC and further gated for CD4^+^. T-cell proliferation was assessed based on the CFSE fluorescence of CD4^+^ T-cells. Right panel: Representative histograms of co-cultured PMN-MDSCs isolated from BALF of PAO-1 infected mice and T-cells at ratios 1:1, 1:2, 1:4, and 1:8 as indicated. **(C)** Ratio-dependent (suppressed) proliferation of CD4^+^ T-cells by co-culturing with neutrophilic MDSCs isolated from BALF of *P. aeruginosa* infected mice is shown. The values are normalized to the proliferation of activated T-cells without addition of MDSCs (T-cells only) as indicated as proliferation index. **(D)** Percentages (top row) and total amounts (bottom row) of neutrophilic MDSCs in BALF, lung, bone marrow, and spleen in acute *P. aeruginosa* infection compared to non-infected control animals. Percentages of neutrophilic MDSCs (PMN-MDSCs) were acquired as % of CD11b^+^Ly6G^+^Ly6C^intermediate^ cells of viable cells (see **A**). Total cell amounts were calculated from cell counts of single cell suspensions from isolated organs/tissues/fluids prior to FACS staining. ^**^p < 0.01. **(E)** Percentages (top row) and total amounts (bottom row) of monocytic MDSCs in BALF, lung, bone marrow, and spleen in acute *P. aeruginosa* infection compared to non-infected control animals. Percentages of monocytic MDSCs (M-MDSCs) were acquired as % of CD11b^+^Ly6C^+^Ly6G^low^ cells of viable cells (see **A**). Total cell amounts were calculated from cell counts of single cell suspensions from isolated organs/tissues/fluids prior to FACS staining. ^*^p < 0.05; ^**^p < 0.01; ^***^p < 0.001.

We characterized the recruitment of neutrophilic and monocytic MDSCs in response to acute *P. aeruginosa* airway infection in different pulmonary (BALF, lung) and extra-pulmonary (spleen, bone marrow) compartments *in vivo* (Figures 1A,D,E). To this end, we analyzed MDSCs based on their (i) phenotypic characteristics (Figure 1A) and their (ii) functional capacity to dose-dependently suppress CD4^+^ T-cell proliferation (Figures 1B,C). Utilizing these approaches we could demonstrate that acute *P. aeruginosa* airway infection lead to an increase of neutrophilic MDSCs in BALF and lung tissue by both percentages as well as total cell numbers and lead to an increase of MDSC percentages in the spleen (Figures 1A,D), whereas the percentages or total cell numbers of neutrophilic MDSCs decreased in the bone marrow compartment (Figure 1D). Percentages of monocytic MDSCs remained unchanged in BALF and bone marrow but increased in lung tissue had a tendential increase in spleen, however to a far lesser extent than neutrophilic MDSCs upon *P. aeruginosa* airway infection (Figure 1E). Total monocytic MDSC numbers also increased in lungs upon *P. aeruginosa* airway infection but remained unchanged in spleen. Collectively, these studies indicate that *P. aeruginosa* airway infection has a substantial effect on MDSCs *in vivo* by triggering the recruitment of neutrophilic MDSCs into the pulmonary compartment and increasing the percentage of neutrophilic MDSCs in the spleen.

### Pseudomonas infection enhances the suppressive capacity of neutrophilic MDSCs

**Figure 2:**
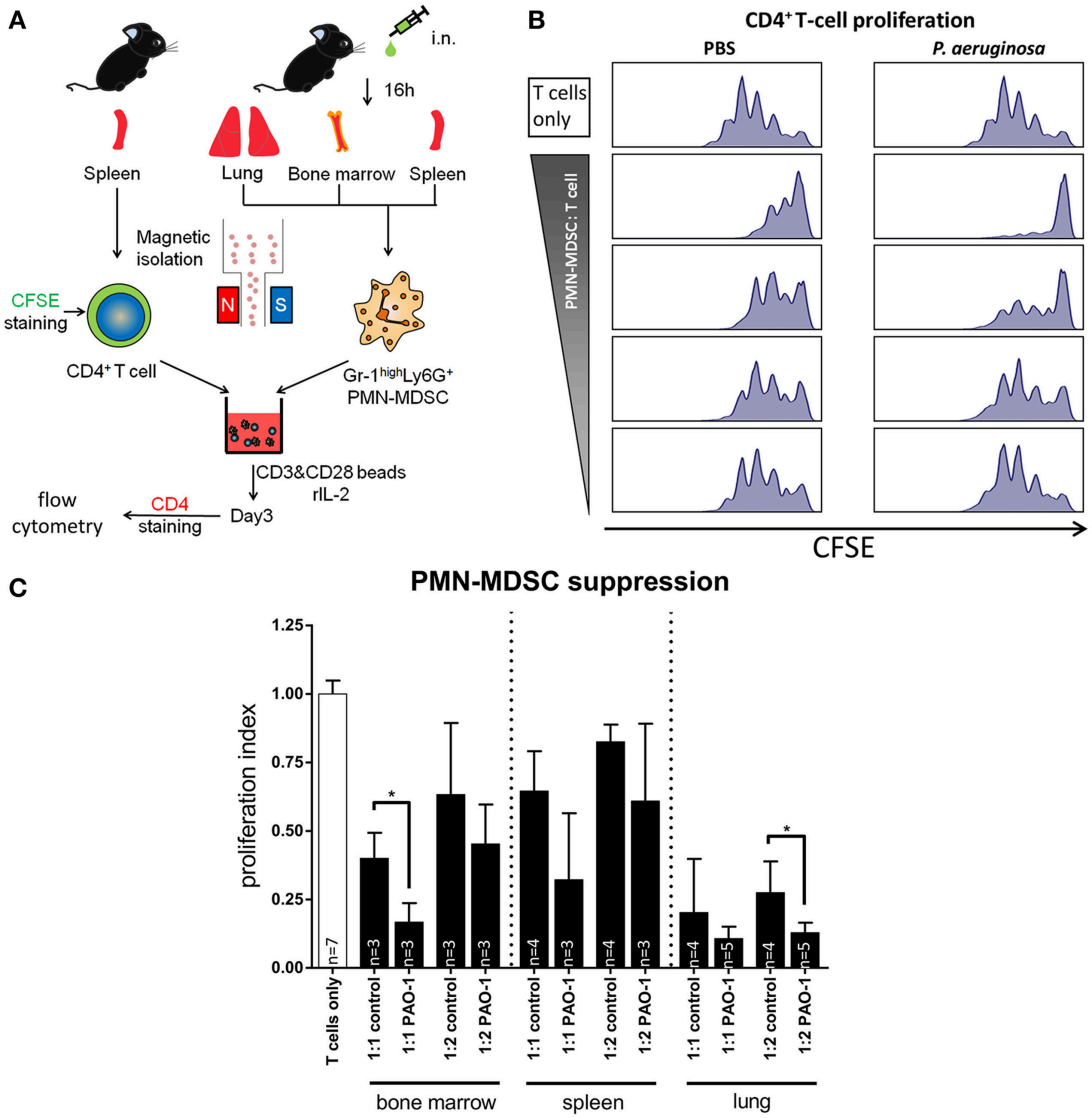
*****Pseudomonas aeruginosa*** airway infection functionally modulates MDSCs**. After *P. aeruginosa* airway infection CD11b^+^Ly6G^+^ neutrophilic MDSCs were isolated from the lung, the bone marrow or the spleens and tested for their potential to suppress polyclonal T-cell proliferation. T-cell suppression assays were performed as described previously (Rieber et al., 2015) using the CFSE method. In brief, isolated CD11b^+^Ly6G^+^ neutrophilic MDSCs were co-cultured for 3 days (37°C, 5% CO_2_) with T-cells (CD4^+^ splenocytes) at MDSC: T-cell ratios 1:1, 1:2, 1:4, and 1:8. T-cells were activated with CD3/CD28-beads and recombinant mouse IL-2. CFSE-fluorescence intensity was analyzed by flow cytometry to determine polyclonal T-cell proliferation. **(A)** Strategy to assess the effect of acute *P. aeruginosa* infection on MDSC-mediated T-cell suppression *ex vivo*. CD4^+^ T-cells were isolated by MACS from splenocytes of C57Bl6/J mice and stained with CFSE. Ly6G^+^ cells were isolated from lungs, bone marrow, and spleens of infected and control C57Bl6/J mice. The isolated cells were co-cultured under T-cell proliferative conditions (with CD3-CD28-coupled beads and rIL-2), collected after 3 days incubation at 37°C 5% CO_2_, stained for CD4 and analyzed by flow cytometry. **(B)** Representative histograms showing CFSE stained control CD4^+^ T-cell proliferation (top) and the suppressed proliferation caused by co-culture with isolated neutrophilic MDSCs (see **A**) from spleens of PBS treated (left) and *P. aeruginosa* infected (right) C57Bl6/J mice with decreasing PMN-MDSC: T-cell ratios (1:1, 1:2, 1:4, and 1:8). Each peak represents one cycle of cell division. **(C)** Graph showing the proliferation of T-cells under T-cell proliferative conditions when co-cultured with suppressive neutrophilic MDSCs isolated from bone marrow, spleens and lungs of PBS treated or PAO1 infected C57BL6/J mice (see **A**). T-cell proliferation was assessed based on CFSE fluorescence of CD4^+^ T-cells. (see Figure 1B). Shown are MDSC: T-cell ratios of 1:1 and 1:2. The values are normalized to the proliferation of activated T-cells without addition of MDSCs (T-cells only), indicated as proliferation index. ^*^p < 0.05.

Next, we sought to dissect whether acute *P. aeruginosa* airway infection not only recruits neutrophilic MDSCs, but also shapes their functional characteristics in terms of suppressing T-cell proliferation. For this purpose, we isolated neutrophilic MDSCs from lungs, bone marrow, and spleens using MACS technology and tested their capacity to suppress polyclonal T-cell proliferation (Figure 2A). These studies demonstrated that spleen-isolated neutrophilic MDSCs suppressed polyclonal T-cell proliferation in a dose-dependent manner (Figure 2B). This effect that was enhanced upon acute *P. aeruginosa* airway infection, but depended on its magnitude and significance on the origin of isolated neutrophilic MDSCs (bone marrow, spleens, or lungs) and the MDSC-to-T-cell ratios applied (Figures 2B,C). In general, neutrophilic MDSCs isolated from the lung had the strongest suppressive capability, followed by neutrophilic MDSCs isolated from bone marrow, while splenic neutrophilic MDSCs showed the weakest suppression of polyclonal T-cell proliferation (Figure 2C). When viewed in combination, these experiments provide evidence that (i) neutrophilic MDSCs in *P. aeruginosa* airway infection functionally dampen T-cell proliferation and that (ii) *P. aeruginosa* airway infection topically (pulmonary) and systemically enhances the suppressive capacity of neutrophilic MDSCs.

### Adoptive transfer of neutrophilic MDSCs has no impact on weight loss and weight recovery in acute pseudomonas infection

**Figure 3:**
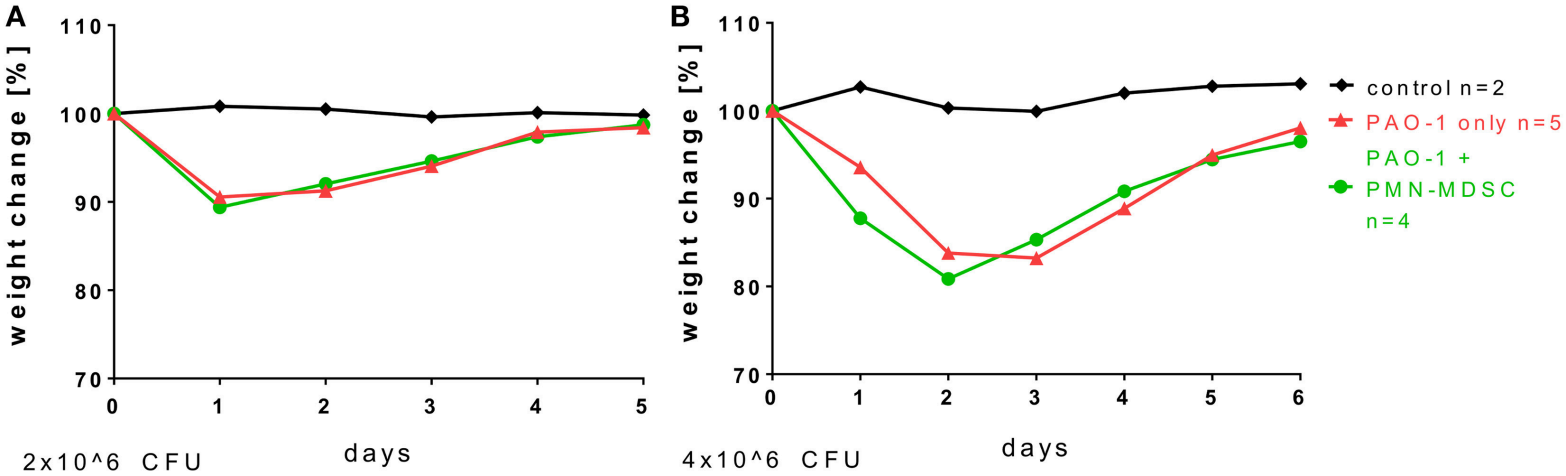
**Adoptive transfer of neutrophilic MDSCs has no impact on weight loss and weight recovery in acute ***Pseudomonas*** infection**. For adoptive transfer experiments, CD11b^+^Ly6G^+^ neutrophilic MDSCs were isolated from the bone marrow of wildtype mice by MACS. Transfer was performed by transferring 8–10 × 10^6^ neutrophilic MDSCs per animal into wildtype mice via lateral tail vein injection. 24 h after the neutrophilic MDSC transfer, mice were infected with doses of 2 × 10^6^ **(A)** or 4 × 10^6^ **(B)** CFU *P. aeruginosa* (PAO1) as described above in detail and weight loss was monitored.

**Figure 4:**
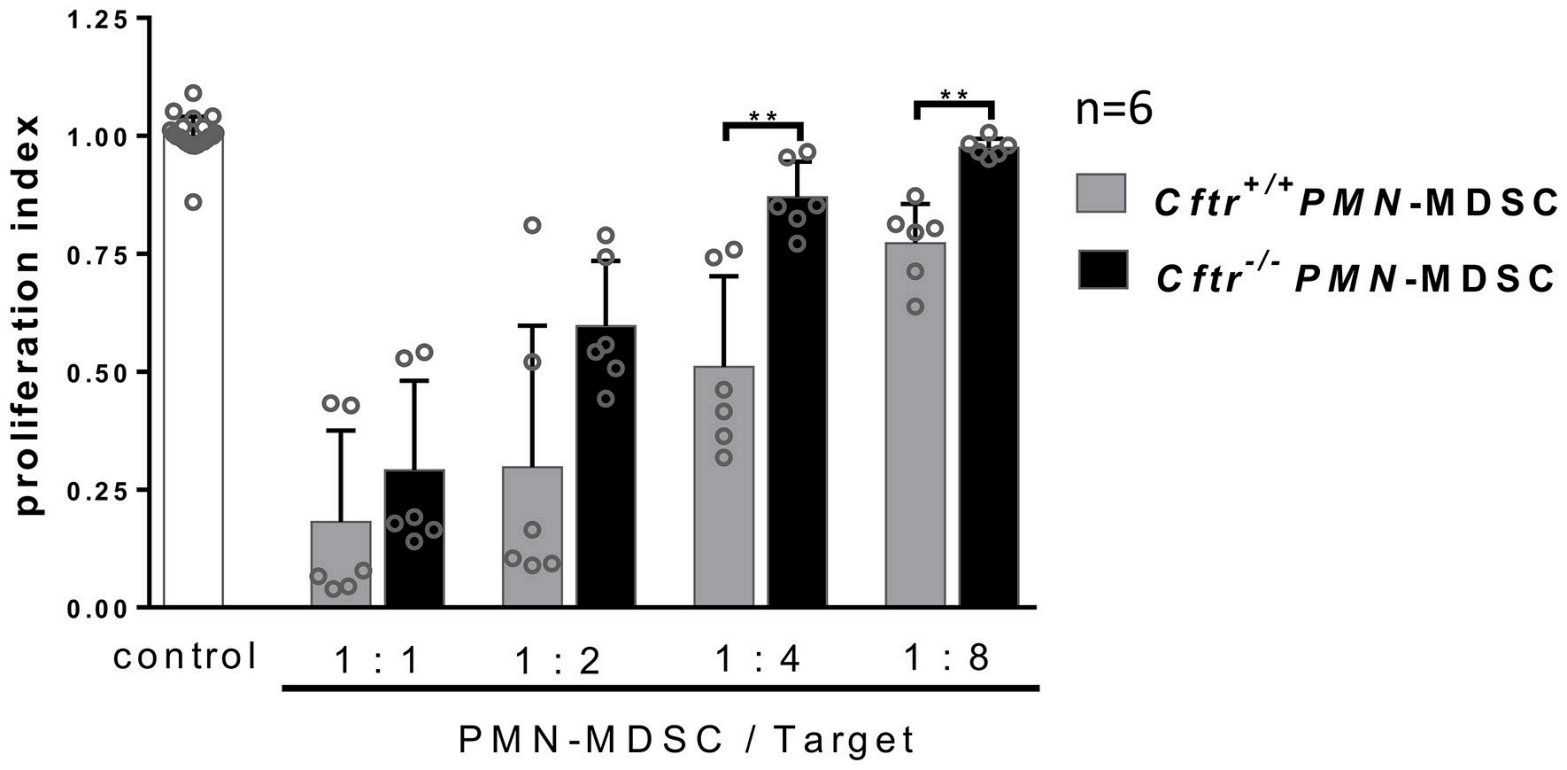
**Role of CFTR in MDSC functionality**. Neutrophilic MDSCs were isolated from 6 days *in vitro* expanded splenocytes in supplemented RPMI and GM-CSF and IL-6 of *Cftr*^+/+^ and *Cftr*^−/−^ mice and tested for their potential to suppress polyclonal T-cell proliferation. T-cell suppression assays were performed as described previously (Rieber et al., 2015) using the CFSE method. In brief, bone marrow isolated *Cftr*^+/+^ or *Cftr*^−/−^ CD11b^+^Ly6G^+^ neutrophilic MDSCs were co-cultured for 3 days (37°C, 5% CO_2_) with T-cells (CD4^+^ splenocytes) at MDSC: T-cell ratios 1:1, 1:2, 1:4, and 1:8. T-cells were activated with CD3/CD28-beads and recombinant mouse IL-2. CFSE-fluorescence of CD4^+^ T-cells was analyzed by flow cytometry to determine polyclonal T-cell proliferation. The values are normalized to the proliferation of activated T-cells without addition of MDSCs (T-cells only), indicated as proliferation index. ^**^p < 0.01.

To assess whether neutrophilic MDSCs bear therapeutic potential in *P. aeruginosa* airway infection, we isolated neutrophilic MDSCs from bone marrow by MACS technology, checked their functionality *ex vivo* in T-cell suppression, adoptively transferred the cells i.v. prior to acute *P. aeruginosa* airway infection and monitored the impact of the adoptively transferred MDSCs on weight loss and weight recovery after acute *P. aeruginosa* airway infection (Figure 3). Two different infection doses were assessed, 2 × 10^6^ (Figure 3A) or 4 × 10^6^ (Figure 3B) CFU. These investigations demonstrated that the adoptive transfer of neutrophilic MDSCs had no significant impact on weight loss or weight recovery upon acute *P. aeruginosa* airway infection at 2 × 10^6^ or 4 × 10^6^ CFU (Figure 3). Both experimental groups successfully cleared the pathogen, as we could not find any residual CFU of *P. aeruginosa* at d5 (2 × 10^6^ CFU) or d6 (4 × 10^6^ CFU) p.i. (data not shown).

### CFTR modulates function, but not recruitment or generation of neutrophilic MDSCs

Since *P. aeruginosa* airway infections play a predominant role in patients with CF, we sought to dissect the role of CFTR in regulating the recruitment and functionality of neutrophilic MDSCs in our experimental systems. For this purpose, we first analyzed whether the absence of functional CFTR has an impact on the recruitment of neutrophilic or monocytic MDSCs into the airways or into non-pulmonary compartments *in vivo* (Supplementary Figures 1, 2). Our data suggest that *Cftr* deficiency has no significant effect on the recruitment of neutrophilic MDSCs (Supplementary Figure 1) in any body compartment *in vivo*, when analyzing *Cftr*^−/−^ mice compared to *Cftr*^+/+^ mice. *Cftr* deficiency also had no significant effect on the recruitment of monocytic MDSCs (Supplementary Figure 2), although the total numbers of monocytic MDSCs were decreased in the bone marrow of *Cftr*^−/−^ mice compared to *Cftr*^+/+^. Next, we assessed whether the lack of CFTR has an effect on the *in vitro* generation of neutrophilic MDSCs (Supplementary Figure 3). Consistent with the *in vivo* recruitment studies, these analyses showed that the lack of CFTR had no effect on the generation of neutrophilic MDSCs *in vitro* (Supplementary Figure 3). Finally, we sought to determine whether the absence of CFTR has a functional consequence for neutrophilic MDSCs in MDSC—T-cell interaction assays. In contrast to the recruitment and generation studies, these functional assays provided evidence that *in vitro* generated *Cftr*^−/−^ neutrophilic MDSCs were impaired in suppressing T-cell proliferation compared to their *Cftr*^+/+^ counterpart cells, most strongly at a 1:4 and 1:8 MDSC: T-cell ratios (Figure 4). In summary, these investigations demonstrated that CFTR has no effect on the recruitment or generation of MDSCs, but regulates, at least partially, MDSC-mediated T-cell suppression.

## Discussion

MDSC generation and functionalities have been studied thoroughly in several types of cancer (Filipazzi et al., 2012; Bruchard et al., 2013; Wesolowski et al., 2013; Youn et al., 2013; Diaz-Montero et al., 2014; Draghiciu et al., 2015; Marvel and Gabrilovich, 2015), yet their regulation and functional role in infectious disease conditions remained poorly defined. Here we studied the role of MDSCs in the setting of airway *P. aeruginosa* infection, which is highly relevant for several human diseases, particularly CF, COPD, ventilation-associated pneumonia and burn-related infections (Hogardt and Heesemann, 2010; Jensen et al., 2010; Williams et al., 2010; Pillarisetti et al., 2011).

These present studies build on previous findings from our group demonstrate that (i) *P. aeruginosa* airway infections in patients with CF were associated with increased percentages of neutrophilic MDSCs in the peripheral blood and that (ii) percentages of circulating neutrophilic MDSCs in *P. aeruginosa* infected CF patients were positively correlated with the lung function outcome (Rieber et al., 2013). Despite these intriguing results, we so far do not have any mechanistic explanation(s) how *P. aeruginosa* infection modulates MDSC recruitment and function and why, unexpectedly, percentages of circulating neutrophilic MDSCs were associated with a beneficial disease outcome in patients with CF lung disease. To dissect this host-pathogen interaction *in vivo*, we systematically investigated the MDSCs in different immune and airway compartments in a well-established model of acute *P. aeruginosa* infection (Munder and Tummler, 2014). Our results here confirmed and extended previous findings obtained from human CF patients that *P. aeruginosa* infections induced MDSC subsets, mainly being neutrophilic MDSCs. *In vivo*, acute *P. aeruginosa* airway infections triggered neutrophilic MDSC recruitment from the bone marrow into bronchoalveolar and pulmonary compartments, where neutrophilic MDSCs were functionally active in suppressing polyclonal effector T-cell responses.

Intriguingly, acute bacterial *P. aeruginosa* airway infections not only recruited neutrophilic MDSCs to the infected airway compartment, but also enhanced, at least partially, their T-cell suppressive potential at a single cell level, surprisingly even at sites distant from the primary site of airway infection, such as the bone marrow. The underlying mechanisms for this phenomenon remain to be solved in the future, but based on our previous finding that neutrophilic MDSCs express Toll-like receptor 5 (TLR5) (Rieber et al., 2013) and its ligand flagellin is shed from *P. aeruginosa* bacteria and can be found in the circulation after *P. aeruginosa* airway infections (Hartl et al., unpublished observation), we tempt to speculate that *P. aeruginosa*-derived pathogen-associated molecular patterns (PAMPs), prototypically flagellin, skew neutrophilic MDSCs systemically, including the bone marrow compartment, to boost their immunosuppressive functionalities, a hypothesis remaining to be tested in future investigations. Overall, the precise *in vivo* kinetics, dynamics and compartmentalizations in the complex setting of *P. aeruginosa* infection remain to be deciphered in future *in vivo* studies.

Besides *P. aeruginosa* infection, we assessed the effect of *Cftr* deficiency on the induction and function of neutrophilic MDSCs, as it is pathophysiological the case for CF patients *in vivo* (Rieber et al., 2013). Our *in vivo* model, however, failed to identify a significant role for *Cftr* in MDSCs homeostasis, at least in our experimental settings, since *Cftr* deficient mice displayed the same amounts of neutrophilic MDSCs as their wildtype littermates in BALF, lungs, spleens, and bone marrow, precluding a major effect of *Cftr*. We also observed no difference on the recruitment and accumulation of neutrophilic MDSCs in BALF and lungs of *Cftr* deficient mice compared to wildtype littermates upon acute pulmonary *P. aeruginosa* infection *in vivo* as well as on neutrophilic MDSC generation *in vitro*.

In contrast to the neutrophilic MDSC-enhancing effect of *P. aeruginosa* infections, *Cftr* deficiency, however, differentially modulated the suppressive capability of *in vitro*-expanded neutrophilic MDSCs. The underlying mechanism requires further investigation, particularly given the complex role of *Cftr* deficiency in driving a hyper-inflammatory micromilieu (Corvol et al., 2003; Livraghi-Butrico et al., 2012) associated with MDSC induction (Rieber et al., 2013; Ballbach et al., 2016). A possible explanation for the diminished suppressive activity of *Cftr*^−/−^ neutrophilic MDSCs could be a scenario where MDSCs may lose their suppressive function via inflammasome activity, as discussed by Koehn et al. (2015).

Monocytic MDSC recruitment was also not affected by *Cftr* deficiency, yet the total amount of cells in bone marrow of *Cftr*^−/−^ mice was diminished compared to *Cftr*^+/+^ littermates, leading to a decreased net total amount of monocytic MDSCs in bone marrow. In-depth characterization of this phenomenon was, however, beyond the scope of this study, as the focus lied mainly on neutrophilic MDSCs, the major CF-relevant MDSC population (Rieber et al., 2013).

Inspired by previous findings from our group that adoptive transfer of neutrophilic MDSCs was protective in an invasive/systemic *in vivo* fungal infection model (Rieber et al., 2015), we tested the potential of adoptively transferred neutrophilic MDSCs to affect the outcome of *P. aeruginosa* infection *in vivo*. However, in our bacterial infection model, the adoptive transfer of neutrophilic MDSCs did not affect the weight loss and/or weight recovery of *P. aeruginosa* infected mice and the bacterial clearance at d5 or d6 p.i. Based on these findings, we speculate that neutrophilic MDSCs have a more substantial impact on invasive/systemic fungal infections compared to compartmentalized *P. aeruginosa* airway infections *in vivo*. The precise mechanisms underlying this difference remain to be defined, but may relate to the fact that in invasive/systemic fungal infections the protective effect of MDSCs was conferred by MDSCs adoptively transferred i.v., the same route as fungal pathogens were inoculated, whereas in contrast in the case of *P. aeruginosa* airway infections, bacteria were inoculated topically into the airways (intranasally) while MDSCs were adoptively transferred i.v. Consequently, in systemic fungal infections both fungal pathogens and MDSCs were in close proximities, whereas in the case of *P. aeruginosa* airway infections, MDSCs would have to actively enter the airway compartment from the circulation.

Our study has several limitations. (i) We were using an acute *P. aeruginosa* infection model for our proof-of-concept MDSC studies; yet a chronic infection/ colonization model, which is much harder to standardize and read-out, would resemble the situation of CF patients more closely; a task for future investigations; (ii) our studies were unable to identify the molecular mechanisms by which *P. aeruginosa*/*P. aeruginosa*-derived factors enhance neutrophilic MDSC activity. Preliminary studies failed to demonstrate significant involvements of ROS and IL-10 (data not shown), issues requiring further immunological and biochemical studies; (iii) lymphocytes express *Cftr*, suggesting that *Cftr* in T-cells could affect the functional outcome of MDSC-T-cell interactions in CF lung disease, though preliminary data from our group showed no significant impact of T-cellular *Cftr* on MDSC-T-cell studies (data not shown); (iv) we observed in our suppression assays that beyond CD4^+^ cells, also non-CD4^+^ cells in the fraction of lymphocytes were proliferating. While we have no clear explanation for this observation and its immunological relevance, we speculate that these non-CD4^+^ T-cells may arise due to the high stimulation trigger during our culture conditions. As the population consistently appeared throughout the experiments, we expect no substantial influence on the outcome of our respective read-out studies; (v) data and knowledge on the dynamics and kinetics of adoptively transferred MDSCs *in vivo* are limited. Preliminary investigations showed that neutrophilic MDSCs reach lungs of mice within 24 h after lateral tail vein injection (data not shown). It is further known from previous studies that adoptively transferred MDSCs can be recovered from splenocytes 5 days post-injection (Koehn et al., 2015), yet studies focusing on the kinetics and dynamics of adoptively transferred MDSCs in the pulmonary compartment are lacking to the best of our current knowledge; (vi) Our adoptive transfer study lacks further in-depth read-outs, such as lung inflammation parameters, amounts, and characteristics of parenchymal T-cells or kinetics on pathogen clearance. Since our MDSC adoptive transfer studies did not reveal any impact of MDSCs on morbidity, we did restrict our read-outs to the minimum. Further studies are required to dissect the temporal and spatial *in vivo* kinetics and dynamics of MDSCs and T-cells in the infected pulmonary compartment.

Collectively, when viewed in combination, this study demonstrates that acute *P. aeruginosa* airway infection recruits and functionally modulates neutrophilic myeloid-derived suppressor cells and thereby defines a mechanism by which *P. aeruginosa* undermines host immunity *in vivo* by modulating neutrophilic MDSCs.

## Author summary

Infections with *P. aeruginosa*, an opportunistic gram-negative bacterium, represent a major cause of morbidity and mortality in patients with CF lung disease. Upon airway infection, *P. aeruginosa* activates the innate immune system. However, in spite of a substantial and sustained presence of phagocytic and lymphocytic immune cells at the infected airway compartment, the host is not able to efficiently eliminate *P. aeruginosa* in CF. The underlying immune mechanisms remained poorly understood. Myeloid-derived suppressor cells (MDSCs) are anti-inflammatory innate immune cells that potently suppress T-cellular immune responses. We showed in a previous study that neutrophilic MDSCs accumulate in patients with CF infected with *P. aeruginosa* and correlate with lung function in those patients. Here, we demonstrate that *P. aeruginosa* airway infection recruits and functionally activates neutrophilic MDSCs and thereby define a mechanism by which *P. aeruginosa* airway infection undermines host immunity by modulating neutrophilic MDSCs *in vivo*.

## Author contributions

HÖ, BZ, CS, and PV performed *in vitro* and *in vivo* experiments. CS contributed to reagents and analysis tools. MC and NF performed *in vivo* experiments. DH, AH, HÖ, and NR designed the study, supervised experiments, and wrote the manuscript. HÖ and DH analyzed the data.

## Funding

This work was supported by the German Research Foundation (RI 2511/2-1 to NR, Deutsche Forschungsgemeinschaft, Emmy Noether Programme HA 5274/3-1 to DH, the CRC/SFB685 to DH). The funders had no role in study design, data collection and analysis, decision to publish, or preparation of the manuscript.

### Conflict of interest statement

The authors declare that the research was conducted in the absence of any commercial or financial relationships that could be construed as a potential conflict of interest.
