# Supplementary material for: Pseudomonas aeruginosa Airway Infection Recruits and Modulates Neutrophilic Myeloid-Derived Suppressor Cells
Source: Front Cell Infect Microbiol. 2016 Nov 29;6:167. doi: 10.3389/fcimb.2016.00167 (PMC5126085; doi:10.3389/fcimb.2016.00167)
Supplement: Supplementary file 1 [file Image1.pdf]

# Supp Figure 1

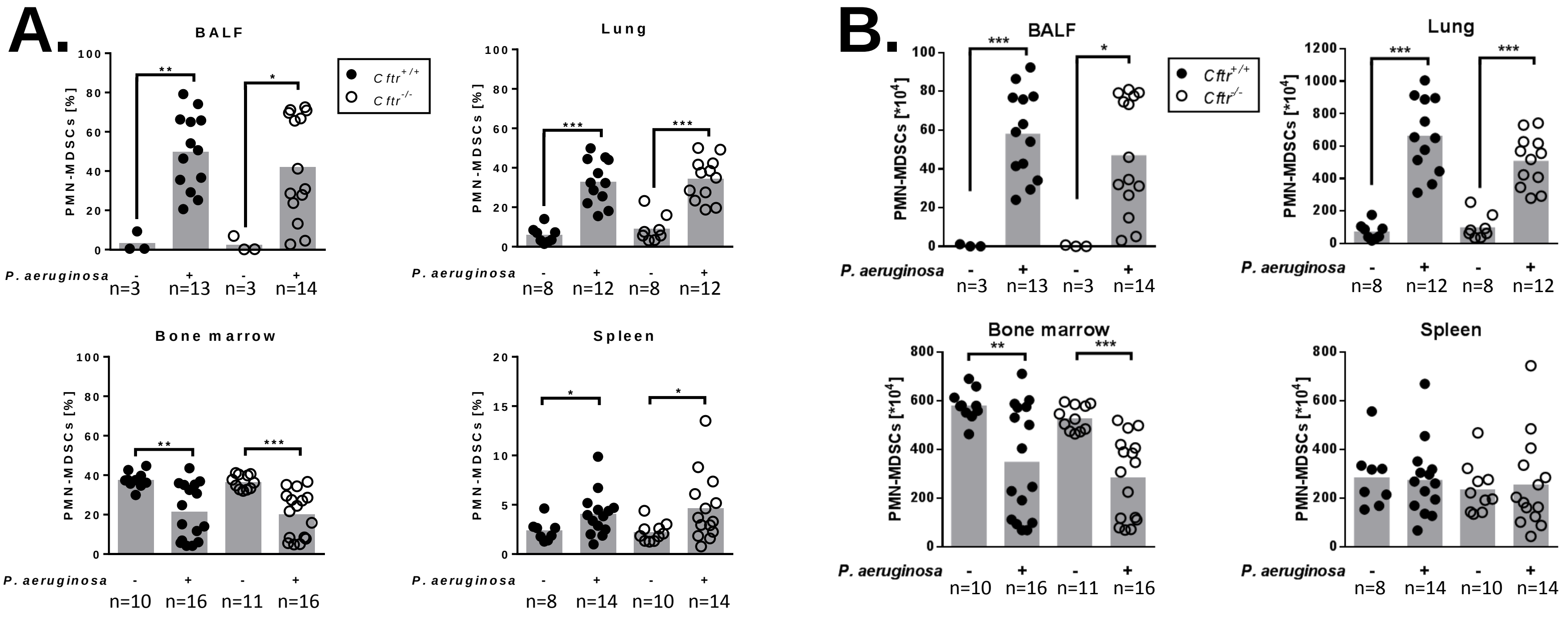


**Fig S1 Role of CFTR in PMN-MDSC recruitment**

Percentages (A) and total cell amounts (B) of PMN-MDSCs in BALF, lung, bone marrow and spleen 16 h after acute *P. aeruginosa* infection in *Cftr*$^{+/+}$ and *Cftr*$^{-/-}$ mice compared to PBS treated mice. Percentages were acquired as % of Ly6G$^{+}$CD11b$^{+}$Ly6C$^{intermediate}$ cells of viable cells (see Fig 1A). Total cell amounts were calculated from cell counts of single cell suspensions from isolated organs/tissues/fluids prior to FACS staining. Filled circles represent *Cftr*$^{+/+}$ while clear circles represent *Cftr*$^{-/-}$ mice. Each circle represents a biological replicate, n numbers are indicated under each bar. For details see methods. *$p < 0.05$; **$p < 0.01$; ***$p < 0.001$
