# Supplementary material for: Pseudomonas aeruginosa Airway Infection Recruits and Modulates Neutrophilic Myeloid-Derived Suppressor Cells
Source: Front Cell Infect Microbiol. 2016 Nov 29;6:167. doi: 10.3389/fcimb.2016.00167 (PMC5126085; doi:10.3389/fcimb.2016.00167)
Supplement: Supplementary file 3 [file Image3.pdf]

Supp Figure 3

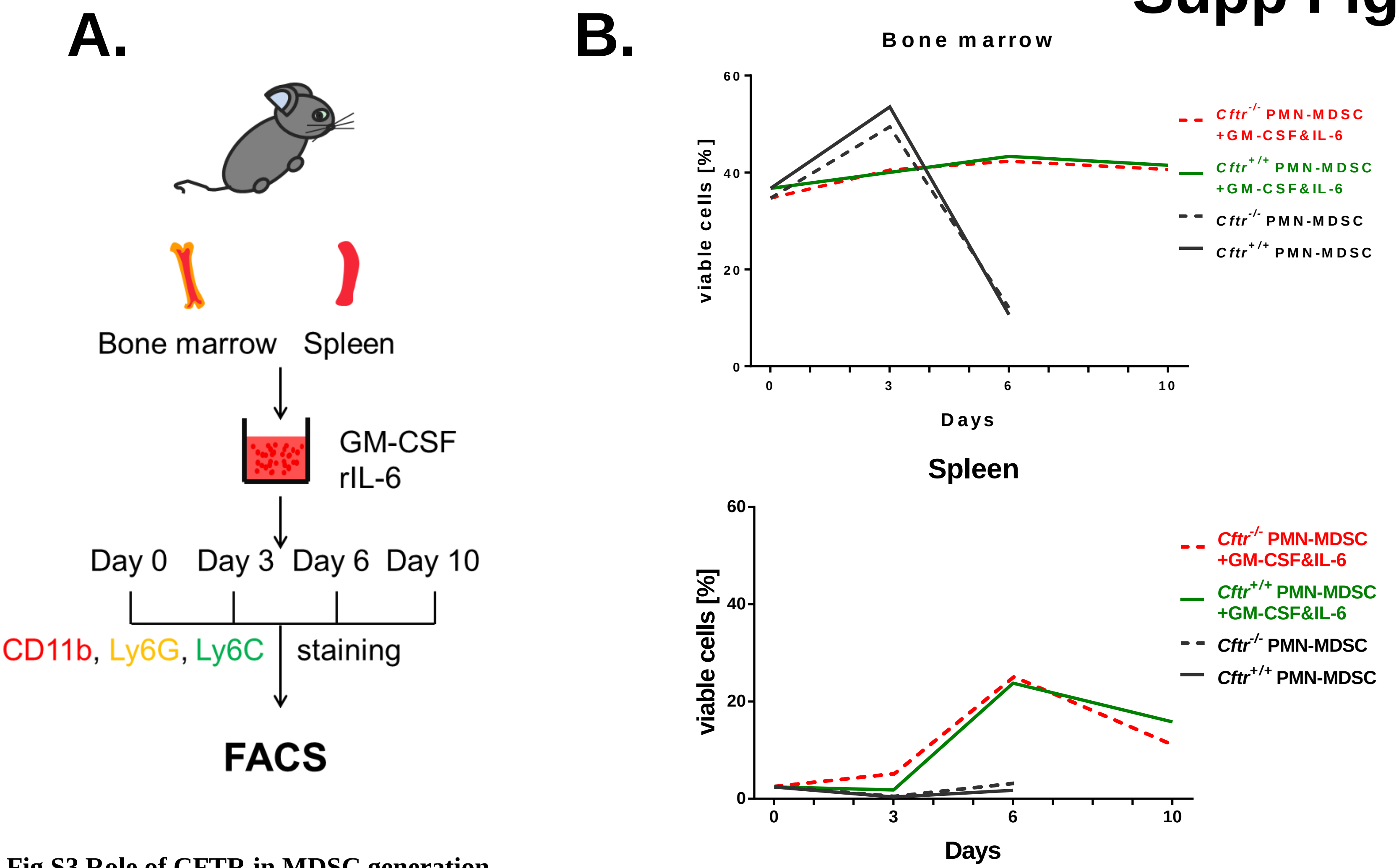

Fig S3 Role of CFTR in MDSC generation

(A) MDSC generation approach. Bone marrow cells and splenocytes were isolated from *Cftr*$^{+/+}$ and *Cftr*$^{-/-}$ mice and cultured in RPMI with supplements (for details see methods section). MDSC generation was induced by addition of 40 ng/ml GM-CSF and 40 ng/ml IL-6. Cells were either fed with fresh media and cytokines on d3 and d6 or collected and analyzed by flow cytometry at d3, d6 and d10.

(B) Percentages of Ly6G^{+}CD11b^{+}Ly6C^{im} cells at d0 (n=6), d3 (n=1), d6 (n=6) and d10 (n=3) generated from *in vitro* expanded *Cftr*$^{+/+}$ and *Cftr*$^{-/-}$ bone marrow cells (top) and splenocytes (bottom).
